# A possible role for VPS13-family proteins in bulk lipid transfer, membrane expansion and organelle biogenesis

**DOI:** 10.1242/jcs.259357

**Published:** 2022-03-10

**Authors:** Thomas J. Melia, Karin M. Reinisch

**Affiliations:** 1 Department of Cell Biology, Yale School of Medicine, New Haven, CT 06520, USA; 2 Aligning Science Across Parkinson's (ASAP) Collaborative Research Network, Chevy Chase, MD 20815, USA

**Keywords:** Membrane contact sites, Membrane homeostasis, Protein-mediated lipid transport

## Abstract

At organelle–organelle contact sites, proteins have long been known to facilitate the rapid movement of lipids. Classically, this lipid transport involves the extraction of single lipids into a hydrophobic pocket on a lipid transport protein. Recently, a new class of lipid transporter has been described with physical characteristics that suggest these proteins are likely to function differently. They possess long hydrophobic tracts that can bind many lipids at once and physically span the entire gulf between membranes at contact sites, suggesting that they may act as bridges to facilitate bulk lipid flow. Here, we review what has been learned regarding the structure and function of this class of lipid transporters, whose best characterized members are VPS13 and ATG2 proteins, and their apparent coordination with other lipid-mobilizing proteins on organelle membranes. We also discuss the prevailing hypothesis in the field, that this type of lipid transport may facilitate membrane expansion through the bulk delivery of lipids, as well as other emerging hypotheses and questions surrounding these novel lipid transport proteins.

## Abstract (precis)

**Summary:** We discuss emerging roles of VPS13-family proteins in vesicle-independent bulk lipid transport between organelles, the possible functions of such transport and the underlying molecular mechanisms.

## Introduction

The composition of the membranes surrounding different cellular compartments differs, both in terms of bulk lipids, such as glycerolipids, sphingolipids and sterols, as well as in terms of the phosphoinositide signaling lipids. Lipids are primarily synthesized in the endoplasmic reticulum (ER), then transported from there to other organelles. The textbook view is that vesicular trafficking plays a major role in lipid transport between organelles, but there must also be other mechanisms for lipid transport because vesicular transport is not sufficiently selective to account for the different lipid compositions of the different compartments; because lipid transport along the secretory pathway is slow, too slow for rapid stress responses; and because some organelles, for example mitochondria and peroxisomes, are disconnected from secretory trafficking. Studies during the past decade have made clear that lipid transport proteins at so-called membrane contact sites, where organelles are closely apposed, play key roles in lipid redistribution between compartments (Scorrano et al., 2019). Most previously characterized eukaryotic transporters comprise small modules that extract and solubilize specific lipids from membranes, then shuttle them across the cytosol for insertion into another membrane. Typically, the transport module features a hydrophobic cavity that accommodates a single lipid molecule, shielding it from the aqueous milieu during transport (Wong et al., 2019). Excitingly, a new mode of non-vesicular transport has recently been discovered, in which elongated proteins are proposed to form bridges between organelles at contact sites that allow bulk lipids to flow between organellar membranes, enabling membrane expansion. The lipids are proposed to flow along channels in the proteins that are lined with hydrophobic residues and thus suited to solubilize glycerolipid fatty acid moieties. Here, we review these recent discoveries, discuss how the proposed model of bulk lipid flow could impact organelle membrane expansion and maturation, as well as how these proteins might integrate into a larger lipid mobilization complex, and finally describe a range of new questions raised by these ideas.

## An emerging concept – VPS13-family proteins function in bulk lipid transport

**Fig. 1.**
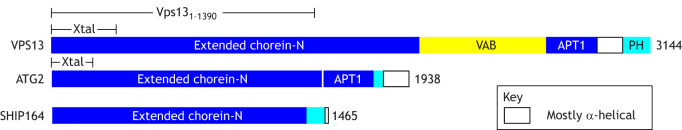
**Domain architecture of characterized proteins in the VPS13 family.** Human VPS13A, ATG2A and SHIP164 are shown. The number of amino acids is indicated for each protein, and the region corresponding to the Vps13_1–1390_ fragment is marked. Xtal, region of the protein for which a crystal structure is available.

The VPS13 proteins, which are conserved across all eukaryotes from yeasts to humans, have been of great biomedical interest because mutations in any of the four human VPS13 genes (*VPS13A*–*VPS13D*) give rise to severe neurological disorders including chorea-acanthocytosis, which shares some phenotypes of Huntington's disease, as well as an early onset form of Parkinson's disease (Lesage et al., 2016; Rampoldi et al., 2001; Ueno et al., 2001). Yet, for a long time the function of these proteins was not known, primarily because they are large (>350 kDa), with little or no homology to previously characterized proteins. The best conserved portion of VPS13 proteins is the ∼120 residues at the N terminus, known as the chorein-N motif. Sequence similarity in the ‘extended chorein-N domain’ immediately downstream of the chorein-N motif is low, but in all VPS13 proteins this segment is highly enriched in β-strand secondary structure (Fig. 1). The VPS13 proteins also contain a VPS13 adaptor binding (VAB) domain, which interacts with receptor-derived peptides (Bean et al., 2018), and an α-helical region and pleckstrin homology (PH) domain at the C terminus, which collectively are important for localization (Bean et al., 2018; Guillen-Samander et al., 2021; Kumar et al., 2018). In addition, the C-terminal portion of VPS13 proteins also contains an APT1 domain (Kaminska et al., 2016). The presence of a chorein-N motif and/or APT1 domain identifies proteins as lipid transporters in the VPS13 family (Kaminska et al., 2016; Neuman et al., 2022; Valverde et al., 2019).

The first clues that VPS13 proteins might be involved in lipid transfer came from studies in yeast. There, gain-of-function mutations in Vps13 rescue dysfunction of ERMES, a multisubunit complex not present in higher eukaryotes that transfers glycerolipids between the ER and mitochondria at contact sites (John Peter et al., 2017). Consistent with a role in lipid transfer, Vps13 also localizes to contact sites (John Peter et al., 2017; Park et al., 2016); for a comprehensive discussion of VPS13 protein localization see (Dziurdzik and Conibear, 2021; Leonzino et al., 2021).

**Fig. 2.**
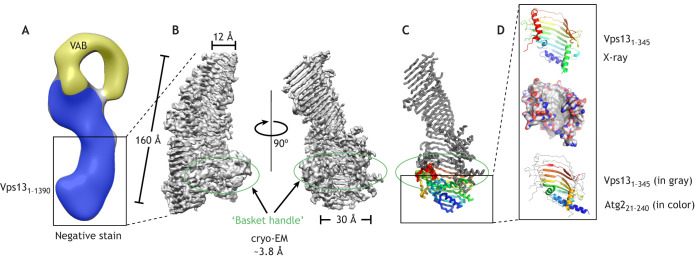
**Structures of VPS13 proteins or parts thereof.** (A) Negative stain reconstruction of intact Vps13 from *Saccharomyces cerevisiae* (∼30 Å resolution) (De et al., 2017). The putative channel is shown in blue, and the VAB is shown in yellow. Box indicates the region shown in B for *C. thermophilum* Vps13. (B) Cryo-EM maps of an N-terminal fragment of Vps13 from the fungus *C. thermophilum*, comprising residues 1–1390, Vps13_1−1390_, at ∼3.8 Å resolution (EMD-21113) (Li et al., 2020), showing an extended β-sheet that resembles a twisted open-ended basket. The basket narrows from ∼30 Å across at its N-terminal end to 12 Å across at the other end. The basket's concave surface likely is lined with hydrophobic residues and can solubilize multiple lipids. An α-helical structure at the wider N-terminal end forms a basket ‘handle’ (circled in green). (C) A ribbons diagram of Vps13_1−1390_; the portion corresponding to the crystallized fragment Vps13_1–345_ (residues 1–345 of Vps13 from *C. thermophilum*) is colored from blue at the N terminus to red at the C-terminal end of the fragment. The basket ‘handle’ is circled in green, and the box marks the region depicted in D. (D) Cartoon representation of Vps13_1–345_, colored from blue at the N terminus to red (top), and a surface representation with carbons colored white, oxygens colored red and nitrogens colored blue (middle) (PDB: 6CBC; Kumar et al., 2018). The cavity is lined with hydrophobic residues. As shown in the structure at the bottom, crystallized portions from the N-termini of Vps13 (in gray) and *Schizosaccharomyces pombe* Atg2 (colored, residues 21–240; PDB: 6A9J; Osawa et al., 2019), including the chorein-N motif at the very N terminus, superimpose closely.

Compelling evidence that VPS13 proteins might themselves be lipid transporters, and furthermore that they may represent a paradigm for an entirely new mechanism of lipid movement, came from biochemical and structural studies. Initial low resolution electron microscopy reconstructions based on negatively stained sample only revealed that intact Vps13 resembles a bubble wand (Fig. 2) that stretches over 20 nm (De et al., 2017). Subsequently, higher-resolution structures of fragments of Vps13 from the fungus *Chaetomium thermophilum* revealed that the long rod-like portion of the bubble wand is the extended chorein-N domain, and further, that this domain adopts a novel protein fold. An atomic resolution crystal structure comprising the first 345 residues of Vps13 (Vps13_1–345_; 3.0 Å) highlights the beginning of a long series of a β-strands later imaged in a near atomic resolution single-particle cryo-electron microscopy (cryo-EM) reconstruction of a longer 160 kDa N-terminal fragment (Vps13_1–1390_; ∼3.8 Å) (Kumar et al., 2018; Li et al., 2020). *In vitro*, this fragment was shown to bind multiple glycerolipids at once (as many as 10 per protein) and to have the ability to transfer such lipids between membranes (Kumar et al., 2018). The structure offers some hints about how this activity might manifest; Vps13_1–1390_ resembles a twisted, open-ended gathering basket (Fig. 2). The basket portion comprises an extended, highly curved β-sheet; α-helices form the ‘handle’ and trim the edges of the basket. The Vps13_1–345_ fragment docks into one end of the ‘basket’. The basket is wide at this end ­– ∼30 Å across (measured between Cα atoms in the peptide chain backbone) – and narrows at the other end to 12 Å across (between Cα atoms). Strikingly, the concave surface of Vps13_1–345_ is lined exclusively by hydrophobic residues, and sequence inspection of β-strands beyond Vps13_1–345_ suggests that the entire cavity of VPS13 proteins is lined with hydrophobic residues, making it plausible that Vps13_1−1390_ and VPS13 proteins can bind many lipids (Kumar et al., 2018; Li et al., 2020). Recently available fold-prediction algorithms (Du et al., 2021; Tunyasuvunakool et al., 2021) further support that the cavity in Vps13_1–1390_ is lined with hydrophobic residues and indicate that the cavity extends even further in intact VPS13 proteins, including into the so-called APT1 domain.

**Fig. 3.**
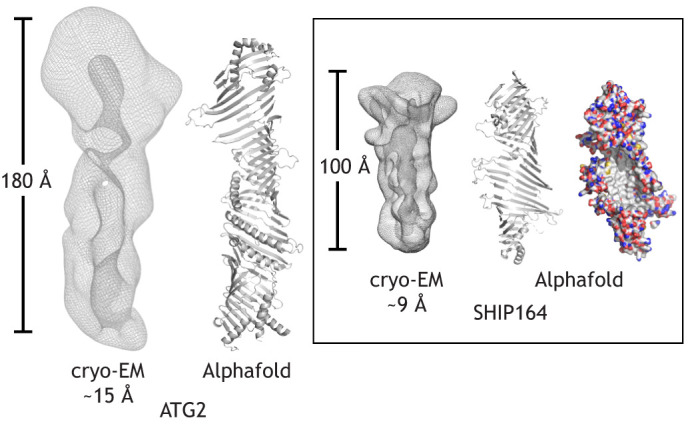
**Architecture of VPS13-family proteins, ATG2 and SHIP164.** Low-resolution cryo-EM maps of human ATG2A (left), generated using data from Valverde et al. (2019), and SHIP164 (right; Hanna et al., 2021 preprint) showing that both feature channels along their lengths. Alphafold (Tunyasuvunakool et al., 2021) predictions are shown in cartoon representation, with positioning of the α-helices only approximate. Structural elements not associated with the ‘basket’ core, including α-helices at the very C termini, are not shown. SHIP164 is also shown as a surface representation, with carbons in white, nitrogens in blue and oxygens red (far right), indicating that the concave surface of the basket is entirely hydrophobic.

Two other chorein-N motif proteins, ATG2 and SHIP164 (also known as UHRF1BP1L), have also been characterized biochemically and structurally (Hanna et al., 2021 preprint; Maeda et al., 2019; Osawa et al., 2019; Valverde et al., 2019). ATG2, but not SHIP164, also has an APT1 domain, but neither of the proteins have the VPS13-specific VAB domain (Fig. 1). In both ATG2 and SHIP164, a short α-helical stretch at their C terminus plays a role in localization (Tamura et al., 2017; Hanna et al., 2021 preprint). A crystal structure for a small N-terminal fragment of yeast Atg2 is available (Osawa et al., 2019), closely matching Vps13_1–345_ in fold (Fig. 2), and low-resolution single-particle cryo-EM reconstructions indicate that the intact proteins are rod-like with a cavity along their length (Hanna et al., 2021 preprint; Valverde et al., 2019). Fold prediction programs (Du et al., 2021; Tunyasuvunakool et al., 2021) suggest that these proteins also feature an extended chorein-N domain comprising expansive, highly curved β-sheets, whose concave surfaces are lined with hydrophobic residues (Fig. 3). Both ATG2 and SHIP164 bind many lipids per protein and each can transfer glycerolipids between membranes *in vitro* (Hanna et al., 2021 preprint; Maeda et al., 2019; Osawa et al., 2019; Valverde et al., 2019).

These proteins are sufficiently long to span the distances between membranes at contact sites (∼10–30 nm, depending on the contact); they can bind multiple lipids at once in a continuous hydrophobic groove, and binding, insofar as it has been characterized (for VPS13, ATG2 and SHIP164 proteins), is not specific for a particular species of glycerolipid (Hanna et al., 2021 preprint; Kumar et al., 2018; Valverde et al., 2019). In addition, VPS13 proteins also promote the formation of contact sites (Kumar et al., 2018), suggesting they might function as tethers that are in contact with both of the apposed organelles. Indeed, both ends of ATG2 can interact directly with membranes *in vitro* (Chowdhury et al., 2018). These observations suggest that this family of proteins may act by a different mechanism than is employed by any previously characterized eukaryotic lipid transporter. Rather than acting as shuttles that ferry single, mostly specific lipids between membranes, these proteins could function as bridges for lipids to move between membranes in bulk. Consistent with a bridge but not shuttle model, mutations in the core of the Vps13 hydrophobic channel that are designed to prevent lipid flow through the core but do not prevent lipid extraction into the channel abrogate Vps13 function (Li et al., 2020). The Vps13_1−1390_ structure also suggests that lipid flow is likely directional because the hydrophobic channel eventually narrows to a width that can only accommodate lipids in single file, as further discussed below.

## What could be the uses of a directional bulk lipid transport system?

### Membrane expansion by bulk lipid transport

Organelles that are not connected to vesicle trafficking pathways, such as the mitochondria and peroxisomes, likely depend strongly upon protein-mediated lipid transport to support their membrane growth and would seem to be prime candidates for the potential bulk lipid transport capabilities of bridge-like proteins. Indeed, *in vivo* observations support the notion that VPS13-family proteins may function to support membrane expansion during organelle biogenesis. For example, metazoan VPS13D plays a role in supplying mitochondria and peroxisomes with ER-derived lipids, and intriguingly, the morphologies of these organelles are affected by VPS13 dysfunction (Anding et al., 2018; Baldwin et al., 2021; Guillen-Samander et al., 2021; Seong et al., 2018). As no mutations have yet been designed to specifically abrogate VPS13D lipid transport apart from its targeting, a direct connection between organelle morphology and VPS13D-mediated lipid delivery still needs to be established.

Unanticipatedly, however, VPS13-family proteins also function in the growth of a number of organelles that until recently have been believed to arise almost exclusively from the fusion of many vesicles, including the autophagosome, the prospore membrane in yeast and the acrosome in sperm. These are all cup-shaped double membrane structures initiated from a small number of Golgi-derived vesicles. Recent studies have demonstrated that the absence of ATG2 specifically leads to a failure to grow autophagosome membranes (Tang et al., 2019) and critically, that the ability of ATG2 to bind and transfer lipids is required to support autophagosome development (Valverde et al., 2019). Similarly, yeast Vps13 and the integrity of its lipid-transfer channel are required for the formation of the prospore membrane (Li et al., 2020), while VPS13B is essential for acrosome formation (da Costa et al., 2020), although whether its lipid-transport capability is required remains unqueried. In addition, SHIP164 has recently been implicated in endosomal growth, although here also it remains unclear whether a lipid-transport capability is required (Hanna et al., 2021 preprint). In each of these cases, vesicles may act as membrane ‘seeds’ – their subsequent growth by either vesicle fusion or bulk lipid transport, or both, may depend upon how closely integrated these organelles are into the secretory system. For example, autophagosome seed formation likely requires a few fusion events to collect Golgi or endosomal vesicles (Kumar et al., 2021; Yamamoto et al., 2012), but the very low density of integral proteins in the mature organelle and the absolute reliance on ATG2 to drive membrane expansion both suggest a fundamental role for bulk lipid transport during this biogenesis event. The relative contributions of protein-mediated lipid transport and vesicle fusion in organelle biogenesis and maturation are still unclear, and they may differ depending on the organelle, pointing to the need of future studies to address these questions.

### Membrane protein dilution by bulk lipid transport

The surface density of both integral membrane proteins and peripherally associated proteins varies significantly across organelles. A new idea suggested by two papers in the field but as-of-yet untested is that very high protein-to-lipid density ratios may restrict some biochemical processes until such time as a VPS13-like protein reduces this density through bulk lipid transport (Hanna et al., 2021 preprint; Sawa-Makarska et al., 2020). For example, cargo, including integral membrane proteins, is highly concentrated in the early stages of endocytosis. During endosome maturation, endosomes undergo local remodeling ahead of cargo sorting into subdomains destined for trafficking to distinct compartments. The precise role of SHIP164 in this process is not yet well understood, but one possibility is that bulk directional lipid transport via SHIP164 contributes to membrane expansion at endosomes in part to decrease the protein-to-lipid ratio in the endocytic vesicles, facilitating more efficient cargo sorting before further trafficking (Hanna et al., 2021 preprint).

Models of autophagosome biogenesis offer an alternative rationale for reducing the protein-to-lipid ratio on cellular membranes. Autophagosomes grow from an uncertain initial membrane (Melia et al., 2020), which several groups have speculated may be individual Atg9-containing vesicles (Atg9 vesicles; Sawa-Makarska et al., 2020; Yamamoto et al., 2012). As the autophagosome grows, enzymes are recruited out of the cytoplasm to drive protein–lipid conjugation events on the growing membrane. Isolated yeast Atg9 vesicles, however, appear to have more than 80% of their surface already occluded by existing proteins, including a high density of Atg9 itself (Sawa-Makarska et al., 2020). Thus, it has been suggested that without approaches to reduce this protein density, recruitment of new cytoplasmic factors and their capacity to drive lipid conjugation might each be sterically inhibited. In this context, an initial bulk deposition of lipid via Atg2-mediated lipid transfer into these vesicles would alleviate this restriction (Sawa-Makarska et al., 2020).

## Bulk lipid transporters and lipid scramblases work in tandem

A condundrum has been that lipid transfer rates measured *in vitro* are much slower than would be required for membrane remodeling or expansion (Reinisch and Prinz, 2021; Wong et al., 2019; Petrungaro and Kornmann, 2019; Prinz and Hurley, 2020). A possible explanation is that *in vivo,* integral membrane proteins collaborate with lipid transporters, perhaps even facilitating the rate-determining step in lipid transport, lipid extraction from the donor membrane. Clues regarding this type of collaboration first came from studies in Gram-negative bacteria, where bridge proteins broadly reminiscent of VPS13-family proteins are components of complexes that transfer lipids between the inner and outer bacterial membranes. Arguably the best characterized bacterial lipid transport system is the lipopolysaccharide transporter, which features a β-sheet ‘bridge’ comprising LptA and LptC that sluices lipopolysaccharides from the periplasmic surface of the inner membrane, where they are made, across the periplasm to the outer bacterial membrane (reviewed in Okuda et al., 2016). The LptA–LptC complex interacts directly with proteins embedded in the inner membrane that load lipids onto the bridge and with proteins embedded in the outer membrane that receive the lipids from the bridge and facilitate delivery of them into another outer membrane complex, LptD–LptE, which in turn transfers arriving lipids across the outer membrane to the outer surface of the bacterium (Okuda et al., 2016).

**Fig. 4.**
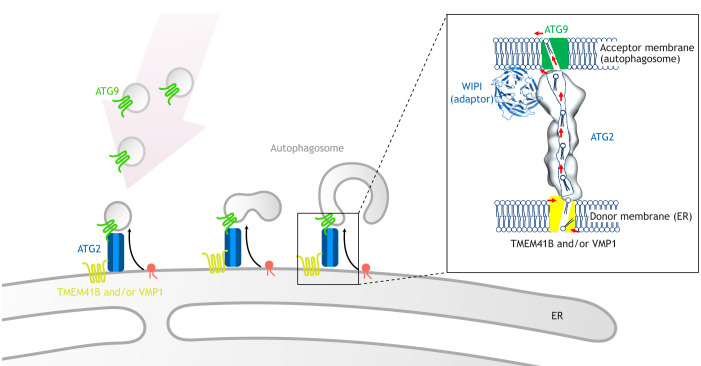
**A current model for ATG2-mediated autophagosome expansion.** Bulk lipid flow from the ER through ATG2 proteins could explain both how the membrane grows from one or a small number of vesicles, and also would necessarily lead to a reduction in the protein-to-lipid ratio in the expanding membrane. As transport is limited to lipids on the cytoplasmic leaflets of the two organelles, scramblases in both membranes would be required to prevent the development of membrane asymmetry. The inset shows the ends of ATG2 associated with scramblases in the ER (TMEM41B and/or VMP1) and the nascent autophagosome (ATG9 proteins) (Ghanbarpour et al., 2021). WIPI adaptor proteins function in recruitment of ATG2 proteins to the autophagosome (PDB: 6KLR; Ren et al., 2020), in analogy with the VAB domain of VPS13 proteins, but most likely do not participate directly in lipid transfer.

Because VPS13-family proteins move lipids between the cytosolic leaflets, rather than both leaflets, of apposed organellar membranes, bulk lipid transfer by a bridge mechanism would destabilize both the donor and the acceptor membranes unless mechanisms exist to re-equilibrate the leaflets of these membranes as lipids are removed or added. For example, several groups have now identified glycerolipid scramblases as integral membrane proteins that are required for, and localize to, sites of autophagosome biogenesis. These include VMP1 and TMEM41B, found complexed in the ER, as well as ATG9 proteins, on the Golgi-derived vesicles that initiate autophagosome formation (Ghanbarpour et al., 2021; Li et al., 2021; Maeda et al., 2020; Matoba et al., 2020). In this way, TMEM41B and VMP1 could re-equilibrate the ER membrane as lipids are removed, while ATG9 re-equilibrates the membrane of the nascent autophagosome as lipids are inserted, allowing for membrane expansion. Intriguingly, although not required for bulk lipid transport in principle, all three scramblases interact directly with the autophagy bridge-like transporter ATG2 (Ghanbarpour et al., 2021). Furthermore, because VMP1 and TMEM41B, but not ATG9, can interact with an N-terminal fragment of ATG2 that includes the chorein-N motif, the orientation of ATG2 at the autophagosome–ER contact site can be inferred. These observations give rise to a model of autophagosome expansion in which TMEM41B and/or VMP1 load lipids from the ER onto ATG2; the lipids then travel through the cytosolic space via ATG2, and are subsequently funneled to ATG9 in Golgi-derived ‘seed’ vesicles (Ghanbarpour et al., 2021) (Fig. 4).

The possibility that VPS13 proteins themselves may link to scramblases is only just beginning to be explored. VPS13A has recently been reported to interact directly with a putative scramblase, XK (Park and Neiman, 2020). Other proteins in the XK family scramble glycerolipids, although such activity has not been reported for XK itself. Supporting the idea that VPS13A and XK collaborate, mutations in either one give rise to similar disease phenotypes (chorea-acanthocytosis and McLeod syndrome, respectively; Park and Neiman, 2020). Since TMEM41B and VMP1 are general scramblases with roles beyond autophagy, we speculate that they may interact with other bridge-like lipid transporters, including possibly the VPS13 proteins themselves, potentially via the well conserved chorein-N motif. Similarly, ATG9 has functions beyond autophagosome biogenesis (Claude-Taupin et al., 2021; Guardia et al., 2021), including possible localization to the developing acrosome (Yefimova et al., 2019). Acrosome biogenesis relies on both autophagy proteins (Huang et al., 2021; Wang et al., 2014) and VPS13 proteins (da Costa et al., 2020), and thus it is tempting to speculate that here or in other membrane expansion events, VPS13 proteins might directly engage ATG9. We anticipate that partner scramblases for VPS13 proteins and related bridge-like transporters will be identified in the near future.

## Key questions moving forward

Recent studies discussed here suggest the existence of non-vesicular bulk lipid transport, and by extension organelle biogenesis, mediated by a partnership of ‘bridge’ proteins and scramblases. We speculate that these complexes cooperate to drive a directional flow of lipid into expanding organelles. However, many questions as to the mechanisms underlying protein-mediated bulk lipid transport remain open, as outlined below.

### What are the mechanisms governing directional lipid transport?

The bacterial lipopolysaccharide transporter system provides one model where the direction of transport is dependent upon the interaction partners of the transporter, including an ATP-driven pump, and its orientation is fixed by engaging with transmembrane proteins on both membranes. What then are the key integral membrane interacting partners for the VPS13 proteins and how do they contribute to VPS13 protein function? Do they support lipid extraction or transfer activities directly? Alternatively, is the energy source for directionality separate from the local protein machinery? Overproduction of lipids on one membrane, for example by lipid synthesis in the ER, could drive an outward lipid flux. Similarly, physical constraints on a receiving membrane might favor lipid influx, for example to relieve the lipid packing defects created by extreme curvature on the autophagosome rim or protein overcrowding in a strained endocytic vesicle. More broadly, we do not yet know whether a net lipid flux is always a necessary outcome. It may be sufficient at some contact sites to simply equilibrate lipid compositions, for example to dilute an otherwise inhibitory lipidic molecule on one surface.

### Can bridge-type transporters be used for selective delivery of specific lipids?

Thus far, *in vitro* biochemistry approaches indicate that VPS13 and ATG2 proteins can extract and transfer a wide range of glycerophospholipids, suggesting that these proteins are not inherently selective, and the structure of a long hydrophobic bridge is most consistent with a non-selective greasy slide. However, recently published work on the bridge-like protein Csf1 in yeast implicates loss of this protein in a failure to move sufficient amounts of phosphatidylethanolamine (PE) from the mitochondria to the ER to support glycosylphosphatidylinositol (GPI)-anchor formation (Toulmay et al., 2022). In the same work, the authors establish that Csf1 directly interacts with the GPI synthesis protein Mcd4 and suggest that Csf1 therefore may be used to funnel mitochondrially derived PE directly into the synthesis enzyme, perhaps implying that PE is the main or only substrate during this event. It will be interesting to see whether Csf1 is naturally selective in lipid-transfer reconstitution assays or if this potential selectivity is a consequence of other factors in the cell. How might selectivity be imposed on a relatively non-selective transporter? One possible mechanism to achieve selectivity is suggested by the coupling of ATG2 to scramblases, thus potentially allowing the lipid cargo of the transporter to be curated by local integral membrane proteins (Fig. 4). In the case of ATG2, these specific scramblases do not exhibit any preference for particular glycerophospholipids as assessed thus far *in vitro* (Ghanbarpour et al., 2021; Li et al., 2021), but it is easy to imagine how integral membrane proteins not yet linked to the VPS13 family – perhaps lipid-specific flippases or lipid biogenesis machinery – could provide an effective way to load a specific lipid into bulk transporters at key contact sites.

### How are bulk lipid transporters distributed throughout the cell?

Contact sites have been described between most pairs of distinct organelles (Scorrano et al., 2019), but the mechanisms governing lipid transport at these sites and the roles of bulk lipid transporters are still being uncovered. Although the early work connecting bridge proteins to lipid transport has focused on VPS13 proteins and ATG2, the family of chorein-domain-containing proteins is growing rapidly to include many other transporters. These include yeast Csf1 and its mammalian ortholog KIAA1109, as well as Fmp27 and Ypr117w (also known as Hob1 and Hob2, respectively; yeast homologs of *Drosophila* Hobbit) (Gomes Castro et al., 2021 preprint; John Peter et al., 2021 preprint; Neuman et al., 2022; Toulmay et al., 2022), each of which associate with ER–mitochondria or ER–plasma membrane contact sites in the cytoplasm, as well as Mdm31 and Mdm32, which function within the mitochondria intramembrane space (Levine, 2019; Miyata et al., 2017). To elucidate whether these proteins are functionally redundant, how they are targeted to specific contact sites and how differences in their structures are used in the cell will require additional work. We anticipate that further characterization of these proteins in the next several years will yield exciting insights regarding both their physiological role and the molecular basis for their function.

## Conclusions

Bridge-like transporters use an entirely new structural paradigm to solve the problem of traversing organelle–organelle contact sites by directly engaging both membranes at once. Here, we have discussed some of the potential consequences and/or advantages of this architecture, including chiefly the possibility of continuous flow across the bridge in support of a bulk lipid transport mechanism. In addition, studies have revealed that these proteins can engage key lipid-mobilizing proteins, such as scramblases, or target directly to lipid metabolism machinery, suggesting that we should imagine these proteins as key cogs in much larger protein complexes.
